# Antiangiogenic Treatment Diminishes Renal Injury and Dysfunction via Regulation of Local AKT in Early Experimental Diabetes

**DOI:** 10.1371/journal.pone.0096117

**Published:** 2014-04-23

**Authors:** Xiaoyan Bai, Xiao Li, Jianwei Tian, Zhanmei Zhou

**Affiliations:** 1 State Key Laboratory of Organ Failure Research, Guangzhou, Guangdong, PRC; National Clinical Research Center of Kidney Disease, Guangzhou, Guangdong, PRC; Guangdong Provincial Institute of Nephrology, Guangzhou, Guangdong, PRC; Division of Nephrology, Nanfang Hospital, Southern Medical University, Guangzhou, Guangdong, PRC; 2 Department of Emergency, Nanfang Hospital, Southern Medical University, Guangzhou, Guangdong, PRC; University of Louisville, United States of America

## Abstract

In view of increased vascular endothelial growth factor-A (VEGF-A) expression and renal dysfunction in early diabetes, we designed a study to test whether VEGF-A inhibition can prevent early renal injury and dysfunction. We investigated the relationship and mechanism between VEGF-A and AKT regulation. *In* *vitro*, VEGF-A small interfering RNA (siRNA) and AKT inhibitor MK-2206 were employed to podocytes and NRK-52 cells cultured in high glucose (30 mM). *In* *vivo*, the antiangiogenic drug endostatin was administered in 12 week-old streptozotocin-induced male Sprague Dawley rats. The levels of VEGF-A, AKT, phosphorylated Ser^473^-AKT, phosphorylated Thr^308^-AKT, nephrin, angiotensin II (Ang II), angiotensin type II receptor 1 (ATR1) were examined using quantitative real-time reverse transcription-polymerase chain reaction (RT-PCR), Western blot analysis and immunohistochemistry. Interactions between phosphorylated Thr^308^-AKT and either nephrin in podocytes or Ang II in renal tubules were studied, respectively, using confocal immunofluorescence microscopy and immunoprecipitation. Silencing VEGF-A in podocytes upregulated phosphorylated Thr^308^-AKT and nephrin. Silencing VEGF-A in NRK-52E cells upregulated phosphorylated Thr^308^-AKT while downregulated Ang II and ATR1. MK-2206 enhanced VEGF-A expression in both podocytes and NRK-52E cells by inhibiting AKT activities. In diabetic rat kidneys, VEGF-A was upregulated and phosphorylated Thr^308^-AKT colocalized with either nephrin in podocytes or Ang II in renal tubules. With the endostatin treatment, the level of VEGF-A decreased while phosphorylated Thr^308^-AKT increased in both glomeruli and renal tubules. Treatment with endostatin upregulated nephrin in podocytes while downregulated Ang II and AT1R in renal tubules. Glomerular mesangial expansion was attenuated by the endostatin treatment, however, differences did not reach statistical significance. Endostatin ameliorated the interstitial fibrosis, urine albumin excretion rate (UAER) and albumin to creatinine ratio. We conclude that phosphorylated Thr^308^-AKT regulates VEGF-A expression by interacting with either nephrin in glomeruli or Ang II in renal tubules. Antiangiogenic treatment improves renal injury and function in early experimental diabetes.

## Introduction

Vascular endothelial growth factor-A (VEGF-A) is a vital proangiogenic protein essential for glomerular development and plays a pivotal role in physiologic and pathologic angiogenesis [1], [2]. In the kidney VEGF-A is predominantly expressed in podocytes, in distal tubules, collecting ducts, and to a lesser extent, in proximal tubules. It promotes the survival, migration, and proliferation of endothelial cells and regulates vessel permeability through paracrine mechanisms. In addition, VEGF-A produced by podocytes may exert an autocrine action, substantially influencing the survival and integrity of the podocyte itself [3], [4]. VEGF-A levels are tightly regulated in the glomerulus and disruption of its expression is associated with alteration in the normal permselective properties of the glomerular filtration barrier and the regulation of the developing glomerular capillary network.

Recent studies have demonstrated an upregulation of VEGF-A and its receptors in experimental animal models of diabetes [5], [6]. Podocyte-specific VEGF-A overexpression in diabetes results in diabetic nephropathy with decreased renal function and ultimately progression to glomerulosclerosis [7]. Whether VEGF-A overexpression plays a causative role in the development of glomerular structural and functional changes in diabetic nephropathy is controversial. For instance, in one study VEGF-A has been implicated in the pathogenesis of kidney disease [8], while other studies have suggested a renoprotective effect [9]–[11]. VEGF-A is also expressed in renal tubular epithelial cells and mediates protein synthesis and renal hypertrophy [12]. However, the role tubular VEGF-A plays in the pathogenesis of diabetic nephropathy has not yet been fully elucidated.

VEGF-A inhibition therapy has had widespread use in treating cancers. Moreover, anti-VEGF-A is also under investigation as a treatment for nonmalignant diseases characterized by disordered angiogenesis, such as rheumatoid arthritis and proliferative diabetic retinopathy [13]. In diabetes, dysregulation of VEGF-A does not occur in isolation. Pathways such as the protein kinase B/AKT pathway have been associated with the regulation of disease progression. Podocyte loss, a characteristic of diabetic nephropathy, may occur through an inability to phosphorylate AKT in response to pathological stimuli [14], [15].

Therefore, in view of high glomerular and tubular expressions of VEGF-A in physiologic circumstances and its upregulation in diabetes, we tested the hypothesis that inhibition of VEGF-A activity by the antiangiogenic drug endostatin prevents renal injury and the onset of early renal dysfunction in a streptozotocin-induced rat model of type 1 diabetes. We investigated the relationships between VEGF-A expression and AKT regulation *in vitro* and during the early stages of diabetes *in vivo*.

## Materials and Methods

### Cell Culture

A conditionally immortalized murine podocyte cell line was cultured as described previously [16] using passages 10 to 18. Rat kidney tubular epithelial cells (NRK-52E; American Type Culture Collection, Rockville, MD) were maintained in Dulbecco’s Modified Eagle’s medium (DMEM)/F-12 containing 10% fetal bovine serum, penicillin (200 U/ml), and streptomycin (200 µg/ml) (Gibco BRL, Grand Island, NY). Both cell lines were cultured in high glucose (30 mM) for one week, grown to 80%–90% confluence and made quiescent by incubation overnight in a serum-free medium.

### Small Interfering RNA Transfection and AKT Inhibitor MK-2206 Treatment

Exponential growth phase cells were plated in 6-well plates at a density of 0.5×10^5^ cells/ml and cultured for 24 hours before experimentation.

Expression of murine VEGF-A was knocked down with small interfering RNA (siRNA) duplexes specifically targeted to murine VEGF-A mRNA. Transfection of siRNA was performed using Oligofectamine (Invitrogen, Carlsbad, CA). The target sequences for VEGF-A mRNA were: 5′-TGCTGTGAAGATGTACTCTATCTCGTGTTTTGGCCACTGACTGACACGAGATAGTACA-TCTTCA-3′, 5′-CCTGTGAAGATGTACTATCTCGTGTCAGTCAGTGGCCAAAACC-3′. Non-targeting siRNA pool (D-001206-13-05; Dharmacon, Fisher Scientific, Pittsburgh, PA) was used as a negative control. Cells were transfected with 1 µg of siRNA in reduced serum medium (OPTI-MEM-I; Invitrogen, Carlsbad, CA) according to the manufacturer’s protocol and harvested 72 hours post transfection. The RNA and protein were extracted and analyzed, respectively. For the AKT inhibition assay, cells were treated with vehicle, dimethyl sulfoxide (DMSO) or 100 nM MK-2206 (sc-364537, Santa Cruz, CA, USA) for 72 hours and then harvested for immunofluorescence staining and Western blot analysis.

### Animal Experiments

The study protocols conform with the Guide for the Care and Use of Laboratory Animals published by the US National Institutes of Health (NIH Publication No. 85-23, revised 1996) and was approved by the Animal Ethics Committee at Nanfang Hospital, Southern Medical University, Guangzhou, China.

Male Sprague Dawley rats, seven weeks of age, weighing between 180 and 200 g, were kept in the Animal Center of Nanfang Hospital, Southern Medical University, Guangzhou, China according to the policy of the Committee for Animal Usage. After overnight fasting for at least 13 hours, the rats were randomized to receive a single intraperitoneal injection of either streptozotocin (65 mg/kg; S0130; Sigma-Aldrich, MO) in 0.1 M citrate buffer (pH 4.5) or 0.1 M citrate buffer. Two days after the injection, plasma glucose concentrations were determined in nonfasting animals using the glucose oxidase method on a glucose analyzer (Accu-check Advantage; Roche, Mississauga, ON). Rats with a glucose level over 16.7 mmol/L were considered diabetic and thus included in the study, followed by treatment with the intraperitoneal administration of endostatin (Shandong Simcere-Medgenn Bio-Pharmaceutical Co., Ltd., Yantai). Plasma and urine glucose level were measured once every two weeks. Four groups containing six animals each were used in the study: normal control rats (NC), normal control rats treated with endostatin (NE), diabetic rats (DMC), and diabetic rats treated with endostatin (DME).

Endostatin was administered at 7.5 mg/m^2^ daily for two weeks, with a one-week interval before the next treatment cycle began. This treatment cycle continued until the rats were euthanized. Serum endostatin level was measured using a commercially available rat endostatin enzyme-linked immunosorbent assay (ELISA) kit (MBS297248, MyBioSource, Inc., CA, USA) at 2, 4, 8 and 12 weeks after treatment.

Twelve weeks after the streptozotocin injection, rats were housed in metabolic cages and urine was collected for the determination of urine albumin excretion rate (UAER) and albumin to creatinine ratio. Blood was collected from the tail vein and plasma samples were prepared for the analysis of glucose, creatinine, blood urea nitrogen (BUN) and VEGF-A. Serum and urine creatinine were analyzed using a Beckman Coulter AU480 Chemistry Analyzer (Beckman, USA). Serum and urine VEGF-A level were measured using rat VEGF Quantikine ELISA Kit (RRV00, R&D Systems Inc., Minneapolis, MN), following the manufacturer’s protocol. Urine albumin was determined with an ELISA kit specific for rat albumin (E111–125, Bethyl Laboratories, Montgomery, TX). All experiments were repeated in triplicate.

At 12 weeks after the induction of diabetes, rats from the four groups were anesthetized with an intraperitoneal injection of pentobarbital sodium (P3761, 30 mg/kg, Sigma-Aldrich, St. Louis, MO) and body weight was obtained. Left kidneys were fixed by retrograde aortic perfusion using phosphate-buffered saline (PBS, pH = 7.4) followed by 10% formalin in PBS for three minutes. Cortex from the left kidneys were placed in the same fixative for 24 hours, rinsed in buffer, dehydrated and embedded in paraffin for histological analysis. The non-perfused right kidney was cut into small pieces, snap-frozen and stored at −80°C for further analysis. Rats were euthanized by exsanguination without any previous intervention following guidelines recommended from the Animal Research: Reporting *In Vivo* Experiments (ARRIVE) (http://www.nc3rs.org.uk/page.asp?id=1357). All efforts were made to minimize suffering.

### Histological Analysis

Five-µm thick paraffin sections were cut, stained with Periodic acid-Shiff (PAS) and Masson’s trichrome staining and used for mesangial and interstitial analyses. Interstitial fibrosis was graded by separately evaluating the tubules and interstitium. Glomerular disease was defined as an increase in PAS staining of the mesangium. Thirty glomeruli and 20 fields of tubules per section were analyzed with a 20× objective lens by two masked independent investigators. Assessment of mesangial expansion was evaluated using a semiquantitative scoring system as follows: 0, no expansion; 1, expansion less than 25%; 2, expansion between 25% and 50%; 3, expansion between 50% and 75%, and 4, expansion greater than 75% of the mesangial area. Assessment of tubulointerstitial injury was performed using Masson’s trichrome stained sections and a semiquantitative scoring system as follows: 0, normal tubulointerstitium; 1, fibrosis less than 25%; 2, fibrosis between 25% and 50%; 3, fibrosis greater than 50% of the observed fields.

### Immunofluorescence and Immunohistochemical Analysis

Snap-frozen right kidney tissues were used for immunofluorescence and perfuse-fixed left kidney tissues were for immunohistochemistry. Podocytes and NRK-52E cells cultured on coverslips were fixed with cold methanol/acetone (1∶1) for 10 minutes at −20°C, followed by blocking with 5% bovine serum albumin (BSA) in PBS (pH = 7.4) for 30 minutes at room temperature before the immunofluorescence staining.

The primary antibodies used were mouse monoclonal anti-VEGF-A antibody (1∶100, ab1316, Abcam, Cambridge, UK), rabbit polyclonal anti-pan-AKT antibody (total AKT antibody, 1∶100, ab8805, Abcam, Cambridge, UK), rabbit polyclonal anti-AKT (phospho Ser^473^) antibody (1∶100, ab66138, Abcam, Cambridge, UK), rabbit polyclonal anti-AKT (phospho Thr^308^) antibody (1∶100, #2965, Cell Signaling Technology, MA, USA), goat polyclonal anti-nephrin antibody (1∶100, sc-19000, Santa Cruz Biotechnology, Inc., Santa Cruz, CA, USA), rabbit polyclonal anti-angiotensin II antibody (Ang II, 1∶100, BOSTER, Wuhan, China), mouse monoclonal anti-angiotensin II type 1 receptor antibody (AT1R, 1∶100, ab9391, Abcam, Cambridge, UK). For immunofluorescence staining, Alexa Fluor 594-conjugated goat anti-mouse IgG and Alexa Fluor 488-conjugated goat anti-rabbit IgG (1∶1000, Invitrogen, Cambridge, MA, USA) were used for secondary antibodies, nuclei were counterstained with 4,6-diamidino-2-phenylindole (DAPI, Sigma-Aldrich, St. Louis, MO) and coverslipped with aqueous mounting medium (CTS011, BD Bioscience, MN, USA). For immunohistochemistry, EnVision Detection Systems Peroxidase/diaminobenzidine (DAB), Rabbit/Mouse kit (K4065, Dako, Carpinteria, CA) was used. Nuclei were counterstained with hematoxylin and coverslipped with Permount mounting medium (00-4960-56, eBioscience, CA, USA).

In each experiment, negative controls without the primary antibody or with an unrelated antibody were done. To avoid interassay variability in immunohistochemical analysis, a kidney sample from each of the four experimental groups was embedded into one paraffin block and thus immunolabelled under the exact conditions.

Immunohistochemical staining was scored semiquantitatively by systematically selecting without bias twenty fields for analysis under 40× objective lens. The staining was graded as follows: 0, no staining; +, mild staining; ++, moderate staining; +++, marked staining; and ++++, strong staining. Images were taken with a BX51 light microscope (Olympus, Japan) or a FV1000-IX81 confocal laser scanning microscope (Olympus). Breast cancer tissues were used as positive controls for VEGF-A, total AKT, and phosphorylated AKT stainings. Kidney tissue was used as an internal positive control for nephrin, Ang II, and AT1R. PBS instead of primary antibodies served as a negative control.

### Laser Capture Microdissection (LCM)

For the studies of protein and mRNA expression, snap-frozen tissues (which had been stored at −80°C) were used. The frozen tissue was cut at 8 µm thickness and placed on a Muster MembraneSlide 1.0 polyethylene naphthalate (PEN) (000757-11, Zeiss, Germany) and was rehydrated briefly in graded alcohols diluted with diethyl pyrocarbonate (DEPC)-treated water. The sections were stained with hematoxylin for 20 seconds, rinsed briefly in DEPC-treated water for 5 seconds, dehydrated in graded alcohols diluted with DEPC-treated water, and air-dried for 20 minutes. The PALM MicroBeam LCM system (Zeiss, Germany) was used for laser microdissection. The laser spot size and beam intensity were adjusted to microdissect pure populations of glomeruli or tubules under direct microscopic observation. For each specimen, 300–400 individual glomeruli or individual tubules were captured sequentially on separate PEN membranes and collected into the caps of eppendorf tubes. For negative controls, caps were placed on the tissue sections in the same way but without activation of the laser pulse.

### Western Blot Analysis and Immunoprecipitation

Cell lysates from cultured podocytes, NRK-52E cells, and microdissected glomeruli or tubules were used for western blot analysis. Lysates from each experimental group were separated in parallel on two 10% denaturing sodium dodecyl sulfate-polyacrylamide gels, transferred onto nitrocellulose membranes, blocked with 5% nonfat milk in 0.1% tris buffered saline with Tween-20 (TBST), and probed using antibodies to VEGF-A (1∶2000), anti-pan-AKT (1∶2000), anti-AKT (phospho Ser^473^) (1∶2000), anti-AKT (phospho Thr^308^) (1∶2000), nephrin (1∶2000), Ang II (1∶2000), and AT1R (1∶2000) at 4°C overnight. After washing, the secondary antibody (horseradish peroxidase-labeled IgG anti-rabbit/mouse antibody, Invitrogen, USA) was used at 1∶3000 dilution for 1 hour at room temperature. The supersignal-enhanced chemoluminescent substrate (Pierce Biotechnology, Inc., Rockford, IL) was applied to the probed membrane and exposed for 10 minutes before the protein bands were visualized on radiograph films (Super Rx, Fuji Photo Film, Tokyo, Japan).

For immunoprecipitation experiments, 1 mg protein from either glomerular or tubular tissue lysate was resuspended in immunoprecipitation buffers (1% Triton X-100, 1% NP-40, 0.5% deoxycholate, 10 mM Tris, pH 8, 0.15 M NaCl, 1 mM EDTA, 50 mM NaF and 1 mM Na3VO4, protease inhibitor cocktail) as described [17], followed by pre-clearing and incubation with anti-AKT (phospho Thr^308^), nephrin, and Ang II.

### Quantitative Real-time Reverse Transcription-polymerase Chain Reaction Analysis

Total RNA from normal control and diabetic rat kidney tissues were extracted using TRIzol reagent (MRC, Cincinnati, OH), and first strand cDNA was synthesized using 2 µg of total RNA treated with Moloney murine leukemia virus reverse transcriptase (Promega, Madison, WI) according to the manufacturer’s instructions. Quantitative real-time reverse transcription-polymerase chain reaction (RT-PCR) analysis was performed in triplicate with Power PCR SYBR Green Master Mix (Applied Biosystems, Carlsbad, CA) using the ABI PRISM 7500 FAST Real-TIME PCR System (Applied Biosystems) with results normalized to β-actin expression. The ΔΔCT method was used to calculate relative expression. Primer sequences used in RT-PCR for rat VEGF-A were (F) 5′-GCAGCGACAAGGCAGACTAT-3′ and (R) 5′-GAGGGAGTGAAGGAGCAACC-3′, rat nephrin were (F) 5′-CATCCTGGCCAACTCGTCCG-3′ and (R) 5′-GGAGTAGGCTGA TCCACCTG-3′, rat AKT were (F) 5′-GGGCCACGGATACCATGAAC-3′ and (R) 5′- AGCTGACATTGTGCCACTGA-3′, rat Ang II were: (F) 5′- CAGACTGCAAGGAGCTTGTCC-3′ and (R) 5′- CTTGGGTCATGGGCATCTTC-3′, rat AT1R were (F) 5′-CATTATCCGTGACTGTGAAAT TG-3′ and (R) 5′-GCTGCTTAGCCCAAATGGTCC-3′, and rat β-actin were (F) 5′-CGCCAGCTCACCATGGATGATGAT-3′ and (R) 5′-TCTCTTGCTCTGGGCCTCGTCG -3′.

### Statistical Analysis

The data are presented as mean ± SD. ANOVA and unpaired t-test were used to test statistical significance. All statistical tests were performed using SPSS 12.0 (SPSS, Inc., Chicago, IL, USA). The significance level was set as P<.05.

## Results

### VEGF-A Silencing Upregulates Phosphorylated Thr^308^-AKT and Nephrin in Podocytes in High Glucose

**Figure 1:**
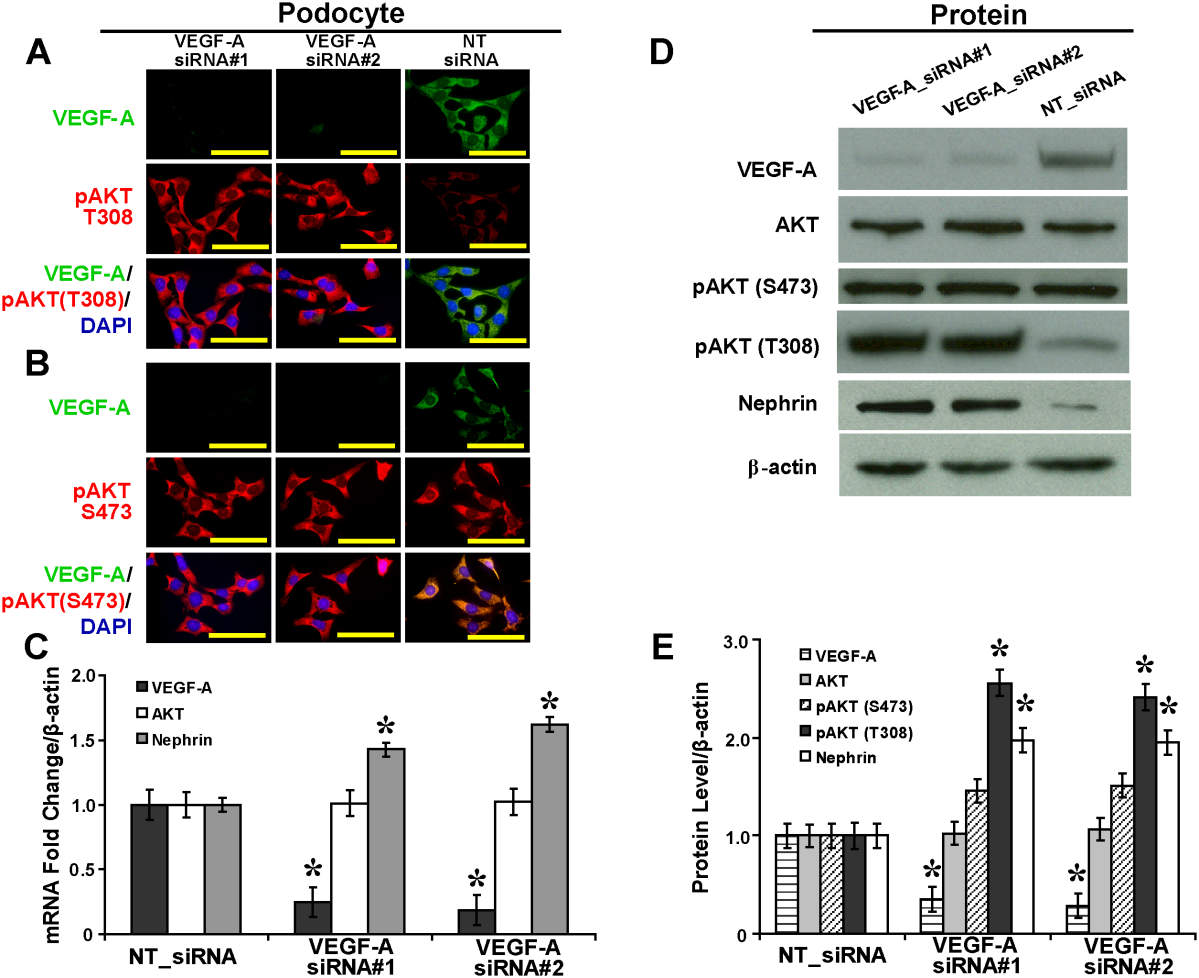
VEGF-A siRNAs cause upregulation of phosphorylated Thr^308^-AKT and nephrin in podocytes in high glucose. (A) Upregulated pAKT (T308) while (B) no effects on pAKT (S473) in podocytes mediated by VEGF-A_siRNA#1 and #2 (immunofluorescence, scale bar 50 mm). (C) Upregulated nephrin mRNA while no effects on total AKT mediated by VEGF-A_siRNA#1 and #2 as shown in quantitative real time RT-PCR. (D) Upregulated protein levels of pAKT (T308) and nephrin, while no effects on total AKT and pAKT (S473) mediated by VEGF-A_siRNA#1 and #2, as shown in Western blot analysis and (E) the relative quantification normalized by β-actin expression (*P<.05 vs. NT_siRNA). VEGF-A: vascular endothelial growth factor-A; pAKT (T308): phosphorylated Thr^308^-AKT; pAKT (S473): phosphorylated Ser^473^-AKT; NT_siRNA: non-targeting small interference RNA.

Upon *in vitro* silencing in podocytes as shown by immunofluorescence (Figure 1A, B), VEGF-A siRNA did not cause significant changes in mRNA or protein levels of total AKT (Figure 1C, D, E). VEGF-A siRNA had no effect on the level of phosphorylated Ser^473^-AKT. In contrast, VEGF-A siRNA significantly upregulated the level of phosphorylated Thr^308^-AKT by 2.48-fold (Figure 1D, E, P<.05), and significantly increased levels of nephrin mRNA by 1.52-fold (Figure 1C, P<.05) and protein by 1.96-fold (Figure 1D, P<.05).

### VEGF-A Silencing Upregulates Phosphorylated Thr^308^-AKT and Downregulates Ang II and AT1R in NRK-52E Cells in High Glucose

**Figure 2:**
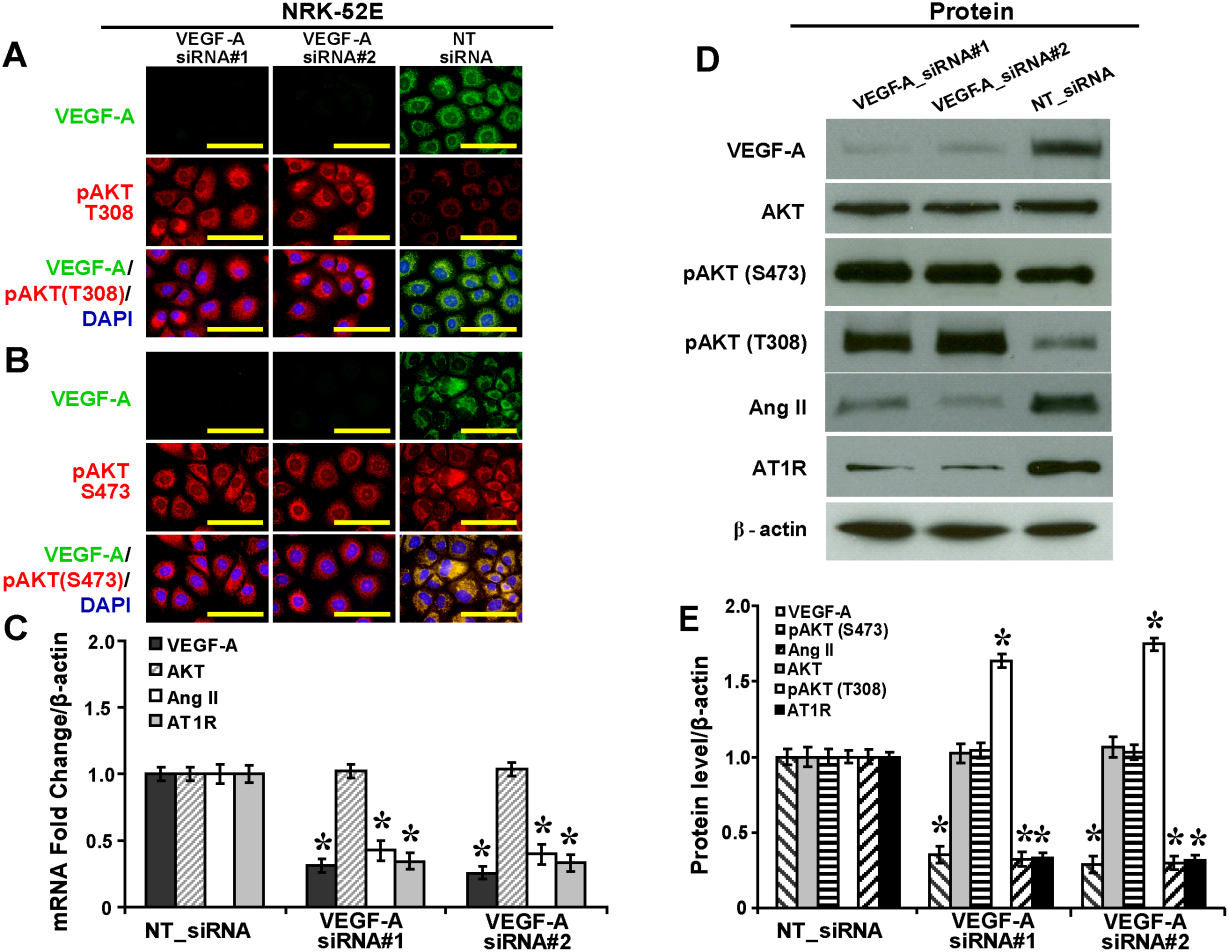
VEGF-A siRNAs cause upregulation of phosphorylated Thr^308^-AKT and downregulation of Ang II and AT1R in NRK-52E cells in high glucose. (A) Upregulated pAKT (T308) while (B) no effects on pAKT (S473) in NRK-52E cells mediated by VEGF-A_siRNA#1 and #2 (immunofluorescence, scale bar 50 µm). (C) Decreased mRNA expressions of Ang II and AT1R while no effects on total AKT mediated by VEGF-A_siRNA#1 and #2 as shown in quantitative real time RT-PCR. (D) Increased pAKT (S473) and decreased Ang II and AT1R, while no effects on total AKT or pAKT (S473) mediated by VEGF-A_siRNA#1 and #2, as shown in Western blot analysis and (E) the relative quantification normalized by β-actin expression (*P<.05 vs. NT_siRNA). Ang II: angiotensin I; AT1R: angiotensin II type 1 receptor; pAKT (T308): phosphorylated Thr^308^-AKT; pAKT (S473): phosphorylated Ser^473^-AKT; NT_siRNA: non-targeting small interference RNA.

Upon *in vitro* silencing in NRK-52E cells as shown by immunofluorescence (Figure 2A, B), VEGF-A siRNA did not cause significant changes in mRNA or protein levels of total AKT (Figure 2C, D, E). VEGF-A siRNA had no effect on the level of phosphorylated Ser^473^-AKT. In contrast, VEGF-A siRNA significantly upregulated the level of phosphorylated Thr^308^-AKT by 1.69-fold (Figure 2D, E, P<.05). For Ang II and AT1R, VEGF-A siRNA significantly decreased levels of mRNA by 59% and 66% (Figure 2C, P<.05) and protein by 69% and 68%, respectively (Figure 2D, E, P<.05).

### AKT Inhibitor MK-2206 Enhances VEGF-A in Podocytes and NRK-52E Cells

**Figure 3:**
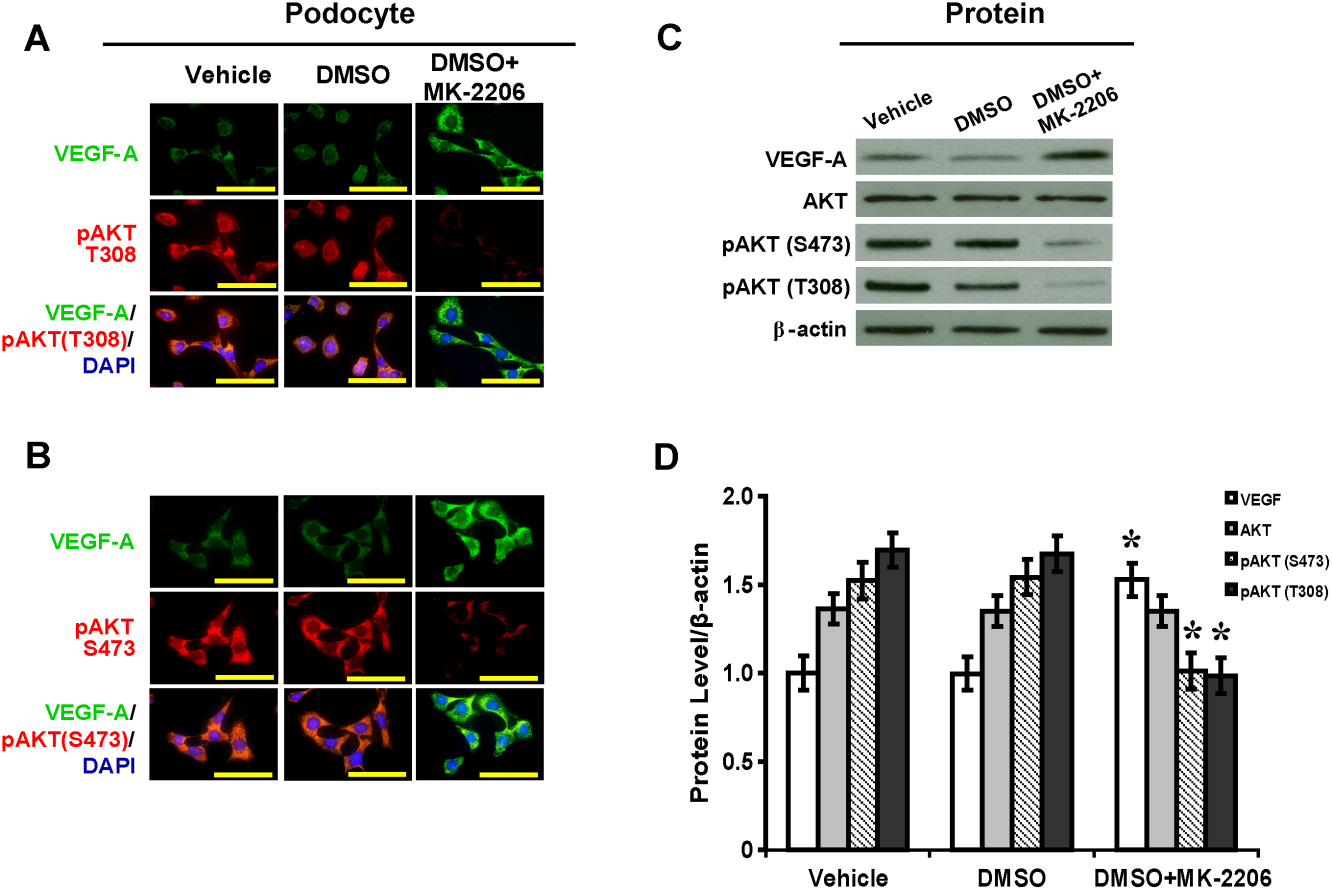
MK-2206 inhibits AKT and upregulates VEGF-A in podocytes. (A) Upregulated VEGF-A and downregulated pAKT (T308) and (B) pAKT (S473) in podocytes with MK-2206 treatment (immunofluorescence, scale bar 50 mm). (C) Inhibition of pAKT (T308) and pAKT (S473) and increased VEGF-A by MK-2206 as shown in Western blot analysis and (D) the relative quantification normalized by β-actin expression (*P<.05 vs. vehicle). DMSO: dimethyl sulfoxide; pAKT (T308): phosphorylated Thr^308^-AKT; pAKT (S473): phosphorylated Ser^473^-AKT.

**Figure 4:**
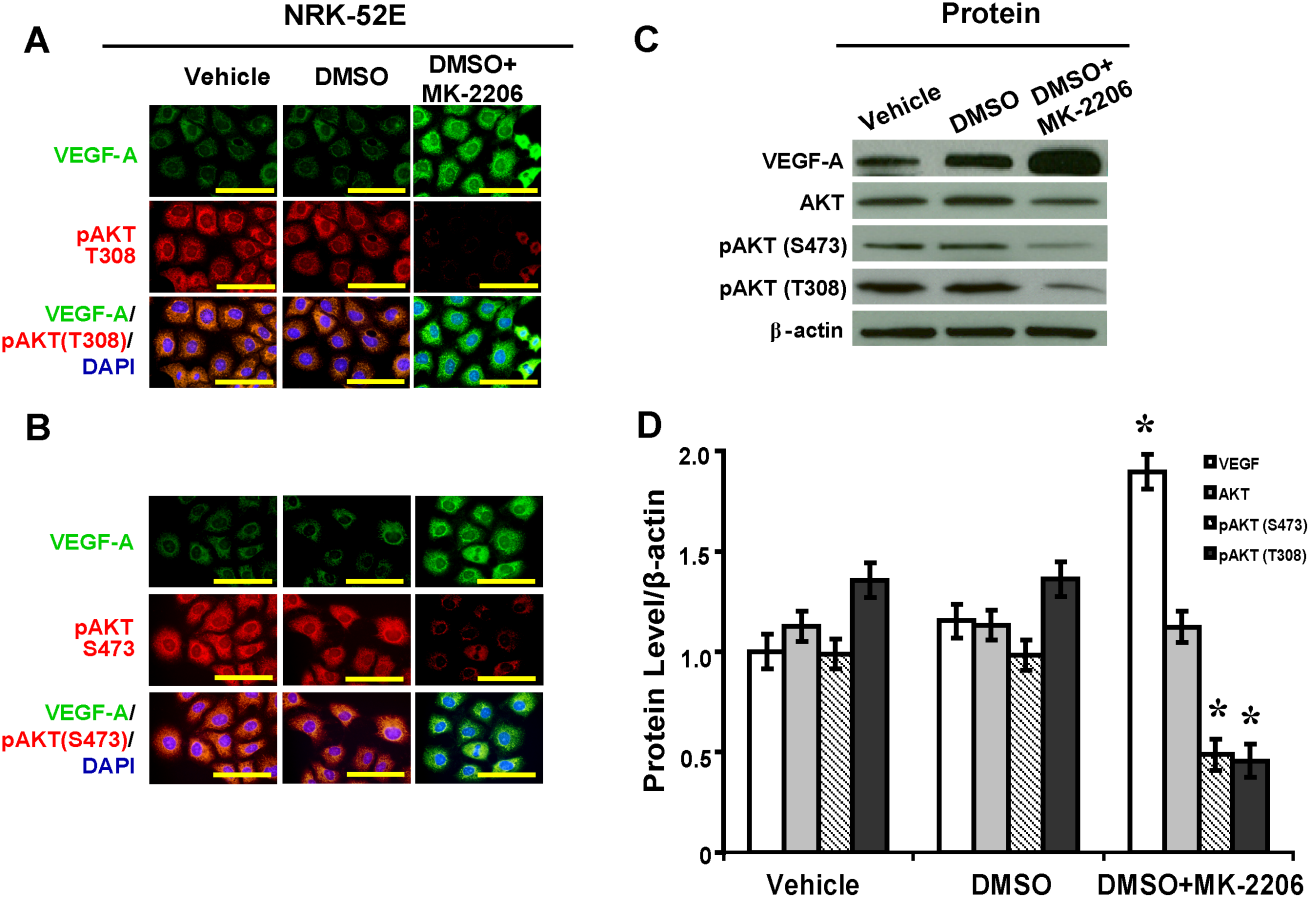
MK-2206 inhibits AKT and upregulates VEGF-A in NRK-52E cells. (A) Upregulated VEGF-A and downregulated pAKT (T308) and (B) pAKT (S473) in NRK-52E cells with MK-2206 treatment (immunofluorescence, scale bar 50 mm). (C) Inhibition of pAKT (T308) and pAKT (S473) and increased VEGF-A by MK-2206 as shown in Western blot analysis and (D) the relative quantification normalized by β-actin expression (*P<.05 vs. vehicle). pAKT (T308): phosphorylated Thr^308^-AKT; pAKT (S473): phosphorylated Ser^473^-AKT.

Treatment with MK-2206 inhibited levels of phosphorylated Thr^308^-AKT and Ser^473^-AKT, resulting in significant increases in VEGF-A levels in both podocytes (Figure 3A, B) and NRK-52 cells (Figure 4A, B). By Western blot and semiquantitative analysis, with MK-2206 treatment in podocytes, levels of phosphorylated Thr^308^-AKT and Ser^473^-AKT were decreased by 41% and 34%, respectively, while VEGF-A was increased by 1.53-fold (Figure 3C, D, P<.05). With MK-2206 treatment in NRK-52 cells, levels of phosphorylated Thr^308^-AKT and Ser^473^-AKT were decreased by 67% and 51%, respectively, while VEGF-A was increased by 1.64-fold (Figure 4C, D, P<.05).

### Antiangiogenic Treatment with Endostatin Improves Renal Function

**Figure 5:**
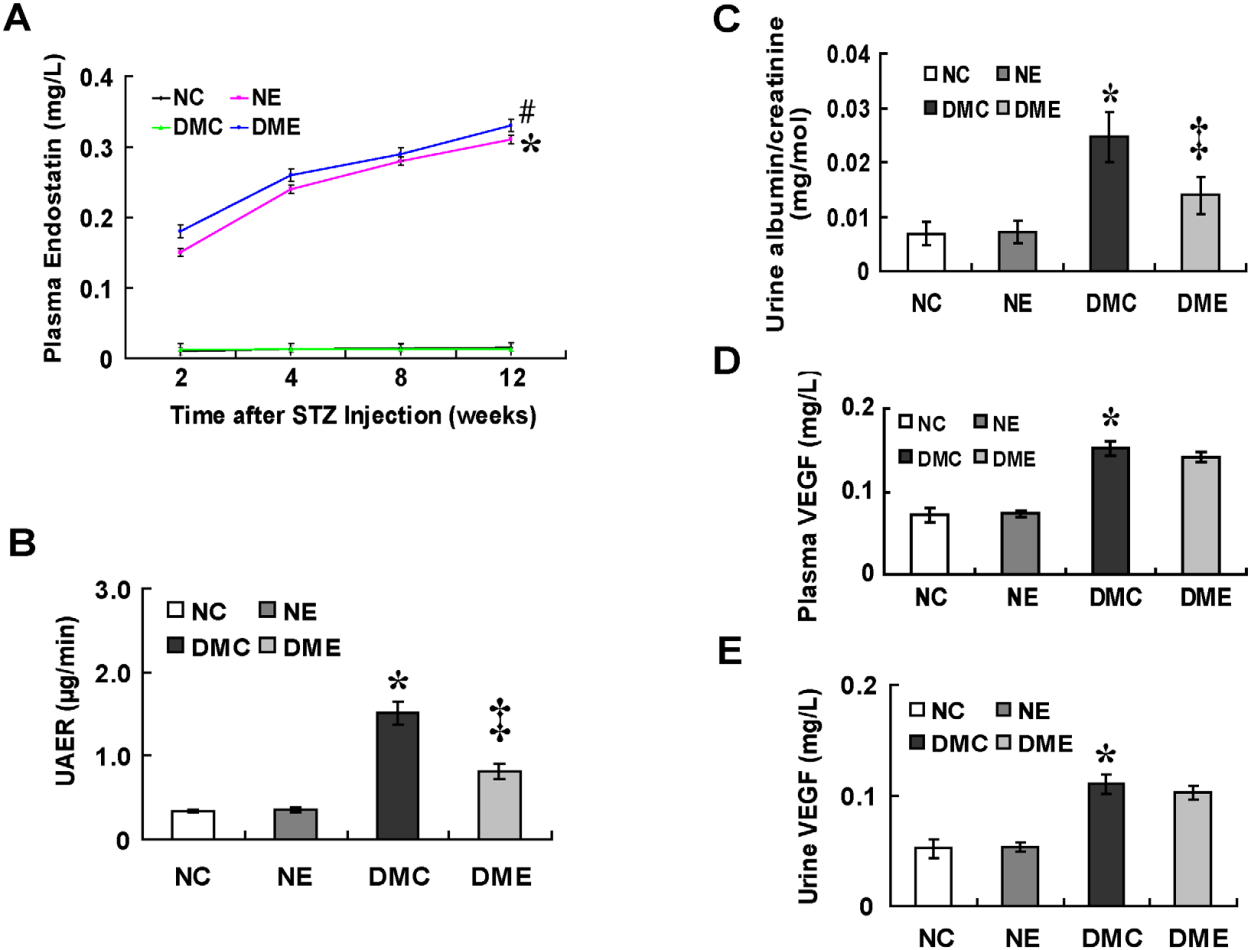
Endostatin treatment improves renal function. (A) Significances in the plasma endostatin level between treated and untreated groups during the endostatin administration (*#P<.05 vs. untreated rats). (B) Increased UAER and (C) albumin/creatinine in DMC group (*P<.05 vs. normal control rats) and attenuated with the endostatin treatment (‡P<.05 vs. diabetic rats). (D, E) Increased plasma and urine VEGF-A levels in diabetic rats (*P<.05 vs. normal control rats) while no effects with the endostatin treatment. NC: normal rats; NE: normal rats treated with endostatin; DMC: diabetic rats; DME: diabetic rats treated with endostatin; UAER: urine albumin excretion rate; albumin/creatinine: albumin to creatinine ratio.

The plasma endostatin value was obtained during the endostatin treatment and there were statistical significances between the treated and untreated groups (Figure 5A, P<.05). UAER and urine albumin to creatinine ratio were increased in diabetic rats compared with normal control rats (P<.05) and were decreased after the endostatin treatment (Figure 5B, C, P<.05). Plasma and urine VEGF-A levels were increased in diabetic rats compared with normal control rats (Figure 5D, E, P<.05) while unaffected by the endostatin treatment. Body weight, kidney weight, plasma glucose and serum creatinine (Scr) were evaluated every two weeks in all groups. The values at 12 weeks after the onset of diabetes are shown in Table 1.

**Table 1:** Metabolic parameters at 12 weeks after the onset of diabetes.

	BW (g)	KW (g)	KW/BW (%)	Plasma Glucose (mmol/L)	Scr (umol/L)
**NC**	282±4	0.83±0.06	0.29	6.57±1.25	78±6
**NE**	282±3	0.81±0.03	0.28	7.27±0.68	74±7
**DMC**	184±5	1.02±0.06	0.56	26.85±3.32	114±7
**DME**	181±3	1.01±0.05	0.56	25.53±2.12	100±2

BW: body weight, KW: kidney weight, Scr: serum creatinine.

### Endostatin Inhibits VEGF-A, Ang II and AT1R and Rescues Phosphorylated Thr^308^-AKT

**Figure 6:**
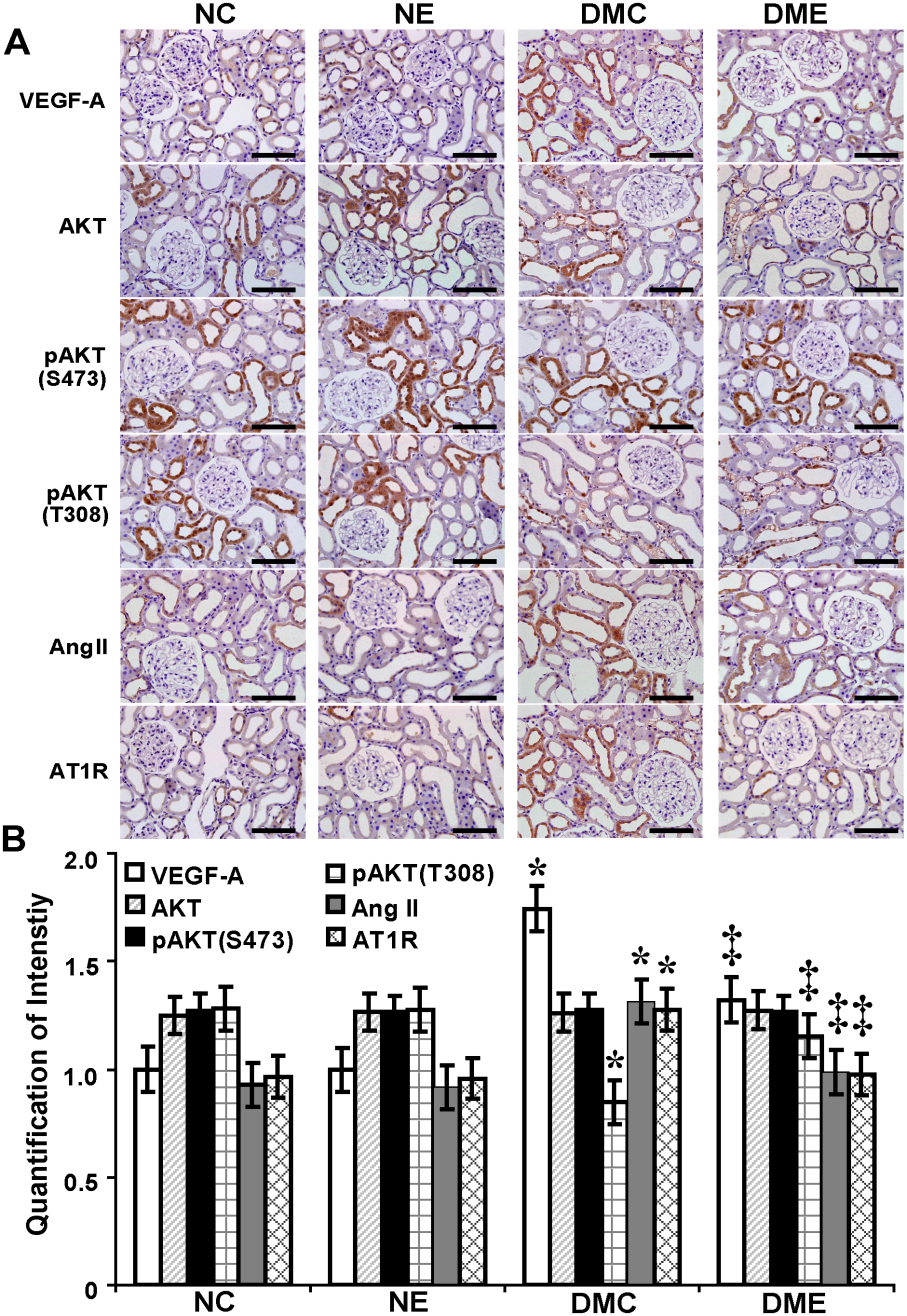
Endostatin inhibits VEGF-A, Ang II and AT1R and rescues phosphorylated Thr^308^-AKT in diabetic kidney tissues. (A) Increased VEGF-A, Ang II and AT1R while decreased pAKT (T308) in group DMC compared with groups NC, NE and DME. No changes in total AKT and pAKT (S473) in the four groups (Immunohistochemistry, scale bar 100 µm). (B) Increased VEGF-A, Ang II and AT1R in group DMC by quantification of staining intensity (*P<.05 vs. normal control rats) and attenuated with the endostatin treatment (‡P<.05 vs. diabetic rats). Decreased pAKT (T308) in diabetic rats (§P<.05 vs. normal control rats) and upregulated with the endostatin treatment (#P<.05 vs. diabetic rats). pAKT (T308): phosphorylated Thr^308^-AKT; pAKT (S473): phosphorylated Ser^473^-AKT.

By immunohistochemistry in kidney tissues and quantification of staining intensity (Figure 6A, B), levels of VEGF-A, Ang II and AT1R were increased by 1.75, 1.42 and 1.32-fold, respectively, and phosphorylated Thr^308^-AKT was decreased by 34% in diabetic rats compared with normal control rats (P<.05). With the endostatin treatment, levels of VEGF-A, Ang II and AT1R were decreased by 24%, 25% and 24%, respectively, and phosphorylated Thr^308^-AKT was increased by 1.36-fold (P<.05). There were no significant changes in levels of total AKT and phosphorylated Ser^473^-AKT before and after the endostatin treatment.

### Interaction between Nephrin and Phosphorylated Thr^308^-AKT Regulates Glomerular VEGF-A

**Figure 7:**
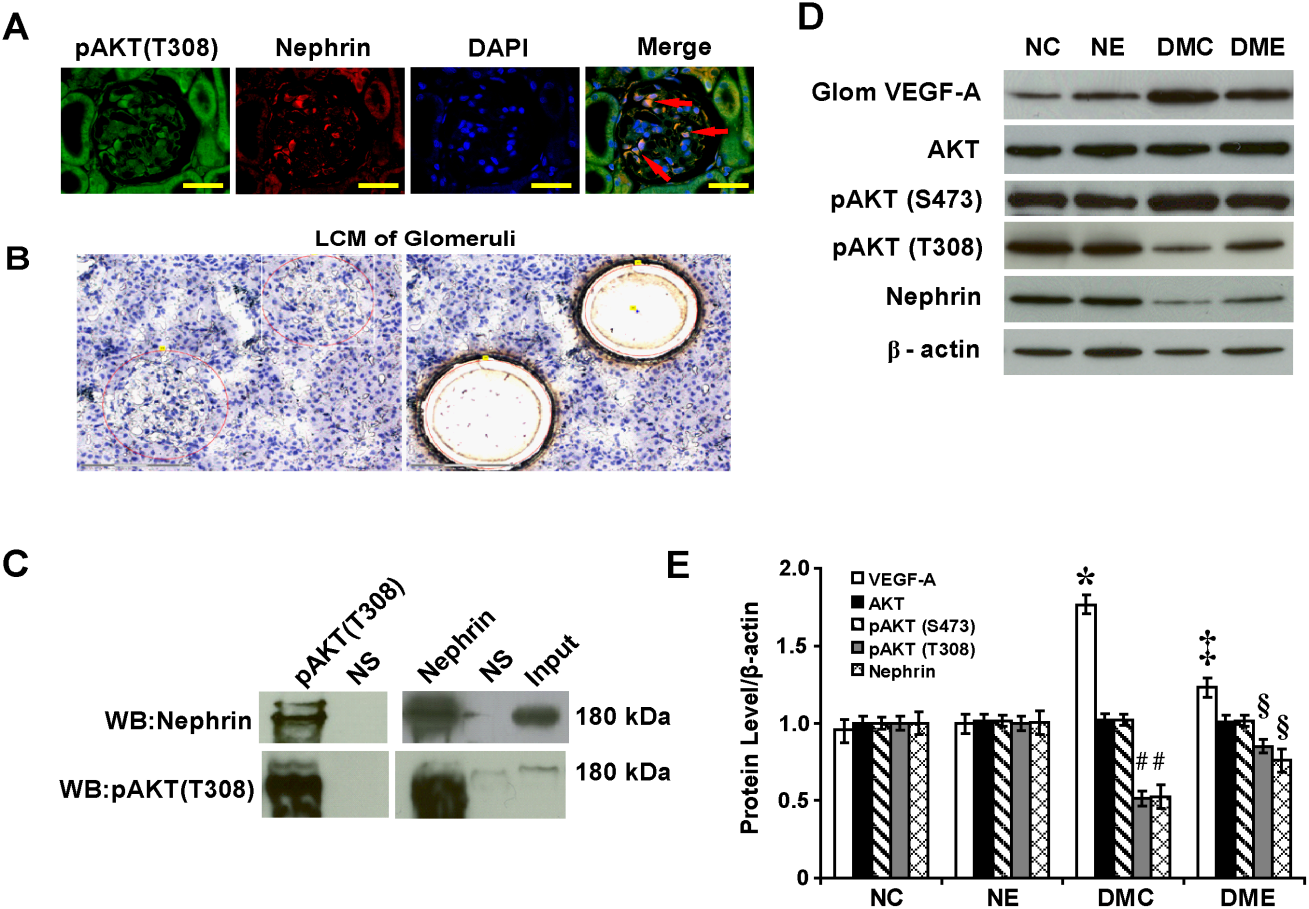
Interaction between nephrin and phosphorylated Thr^308^-AKT regulates glomerular VEGF-A. (A) Colocalization of pAKT (T308) and nephrin in podocytes (arrows) in diabetic rats (immunofluorescence, scale bar 50 µm). (B) Isolated individual glomeruli by LCM. (C) Interaction between pAKT (T308) and nephrin *in vivo* by immunoprecipitation. (D) By Western blot and (E) semiquantitative analysis, upregulated glomerular VEGF-A in diabetic rats (*P<.05 vs. normal control rats) and downregulated with the endostatin treatment (‡P<.05 vs. diabetic rats). Downregulated glomerular pAKT (T308) and nephrin in diabetic rats (#P<.05 vs. normal control rats) and upregulated with the endostatin treatment (§P<.05 vs. diabetic rats). LCM: laser capture microdissection; pAKT (T308): phosphorylated Thr^308^-AKT.

Nephrin co-localized with phosphorylated Thr^308^-AKT in the membrane of podocytes as detected by immunofluorescence (Figure 7A). To analyze the mechanisms of how glomerular VEGF-A was regulated, individual glomeruli were isolated using LCM (Figure 7B). Phosphorylated Thr^308^-AKT interacted with nephrin *in vivo* detected by immunoprecipitation (Figure 7C). By Western blot and semiquantitative analysis, VEGF-A level was increased by 1.77-fold and levels of phosphorylated Thr^308^-AKT and nephrin were decreased by 49% and 48%, respectively, in diabetic rats compared with normal control rats (P<.05). With the endostatin treatment, VEGF-A level was decreased by 30% and levels of phosphorylated Thr^308^-AKT and nephrin were increased by 1.66 and 1.45-fold, respectively (P<.05). There were no significant changes in levels of total AKT and phosphorylated Ser^473^-AKT before and after the endostatin treatment (Figure 7D, E).

### Interaction between Ang II and Phosphorylated Thr^308^-AKT Regulates Tubular VEGF-A

**Figure 8:**
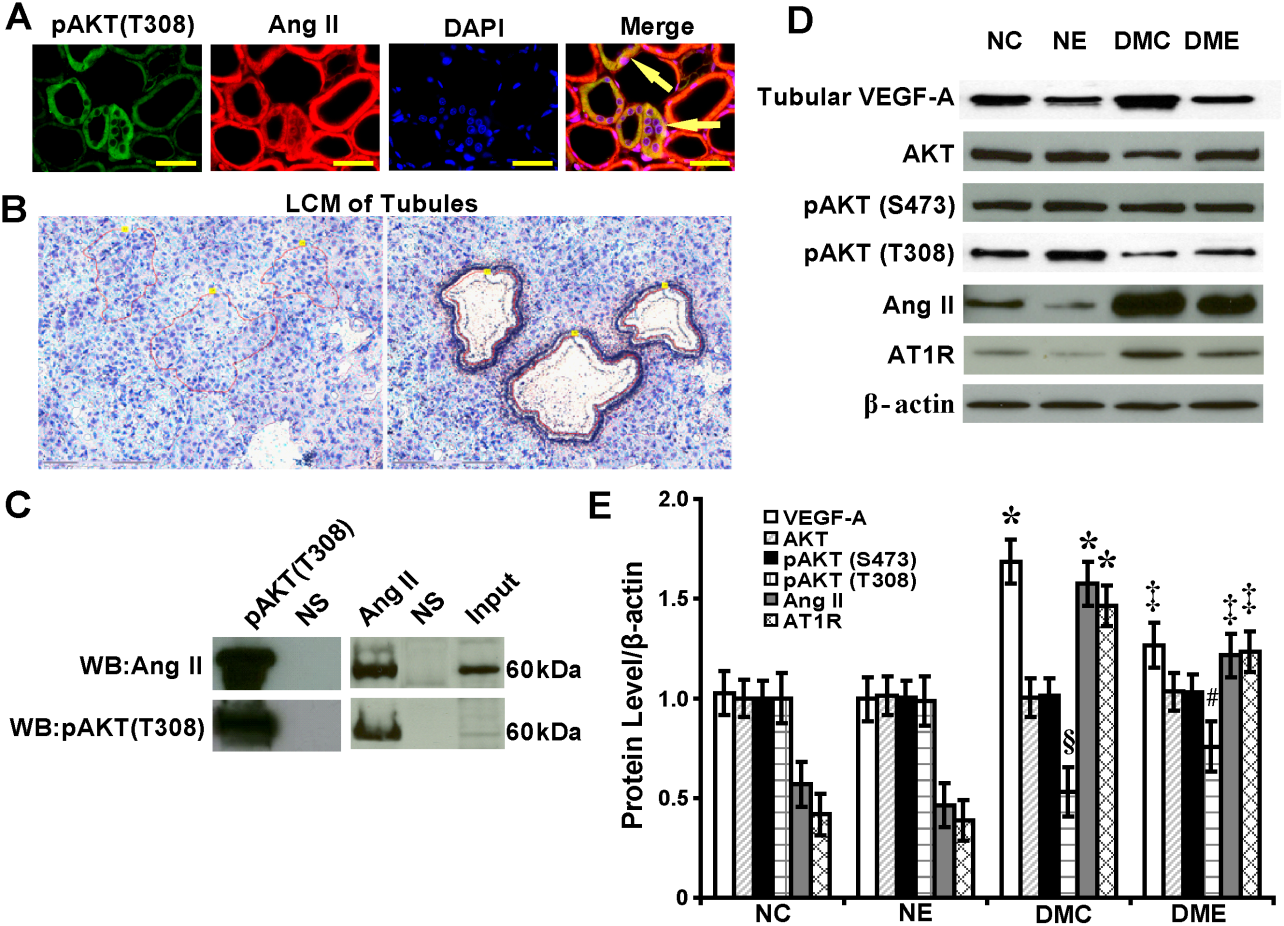
Interaction between Ang II and phosphorylated Thr^308^-AKT regulates tubular VEGF-A. (A) Colocalization of pAKT (T308) and Ang II in renal tubules (arrows) of diabetic rats (immunofluorescence, scale bar 50 µm). (B) Isolated individual tubules by LCM. (C) Interaction between pAKT (T308) and Ang II *in vivo* by immunoprecipitation. (D) By Western blot and (E) semiquantitative analysis, upregulated tubular VEGF-A and Ang II in diabetic rats (*P<.05 vs. normal control rats) and downregulated with the endostatin treatment (‡P<.05 vs. diabetic rats). Downregulated tubular pAKT (T308) in diabetic rats (§P<.05 vs. normal control rats) and upregulated with the endostatin treatment (#P<.05 vs. diabetic rats). pAKT (T308): phosphorylated Thr^308^-AKT.

Ang II co-localized with phosphorylated Thr^308^-AKT in the cytoplasm of renal tubular epithelial cells as detected by immunofluorescence (Figure 8A). To analyze the mechanisms of how tubular VEGF-A was regulated, individual renal tubules were isolated using LCM (Figure 8B). Phosphorylated Thr^308^-AKT interacted with Ang II *in vivo* detected by immunoprecipitation (Figure 8C). By Western blot and semiquantitative analysis, levels of VEGF-A, Ang II and AT1R were increased by 1.69, 1.58 and 1.46-fold, respectively, and phosphorylated Thr^308^-AKT was decreased by 47% in diabetic rats compared with normal control rats (P<.05). With the endostatin treatment, levels of VEGF-A, Ang II and AT1R were decreased by 30%, 31% and 23%, respectively, and phosphorylated Thr^308^-AKT was increased by 1.42-fold (P<.05). There were no significant changes in levels of total AKT and phosphorylated Ser^473^-AKT before and after the endostatin treatment (Figure 8D, E).

### Endostatin Ameliorates Interstitial Fibrosis in Diabetes

**Figure 9:**
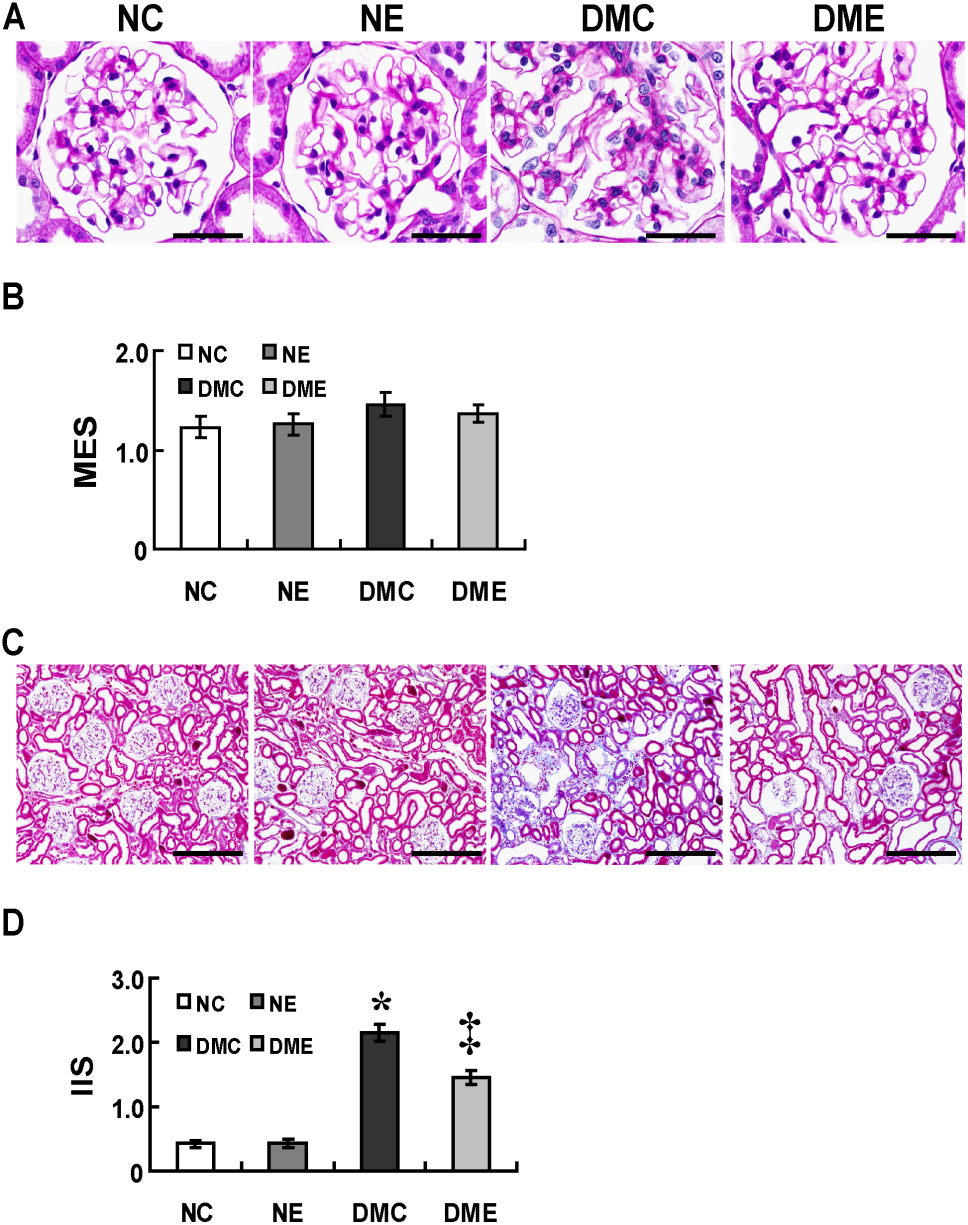
Endostatin attenuates interstitial fibrosis in diabetes. (A) No significant mesangial expansion in group DMC compared with groups NC, NE and DME (PAS, scale bar 50 µm). (B) No significances in the MES of diabetic rats before and after the endostatin treatment. (C) More prominent interstitial fibrosis in group DMC compared with groups NC and NE and attenuated with the endostatin treatment in group DME (Masson’s trichrome stain, scale bar 200 µm). (D) Increased IIS in group DMC compared with groups NC and NE (*P<.05) and decreased with the endostatin treatment in group DME (‡P<.05). PAS: Periodic acid-Schiff; MES: mesangial expansion score; IIS: interstitial injury score.

The treatment with endostatin tended to attenuate the mesangial expansion in diabetic rats, however, differences did not reach statistical significance (Figure 9A, B). In contrast, the interstitial fibrosis was more prominent in diabetic rats compared with normal control rats and was attenuated significantly by the endostatin treatment (Figure 9C, D, P<.05).

## Discussion

Diabetic nephropathy is a common complication of diabetes with increased morbidity and mortality. People have a very high risk for chronic renal failure due to micro- and macrovascular diabetic complications. Diabetic nephropathy is characterized by structural changes in glomeruli including hypertrophy, matrix expansion, basement membrane thickening, formation of nodules, and obsolescence of the capillary tuft. Most of these changes have been shown to be modulated by cytokines such as angiotensin II, nitric oxide, as well as VEGF-A [18].

VEGF-A plays an important role in the regulation of glomerular growth and function [19]. It is increased in several cell types and tissues by high glucose concentrations, such as vascular smooth muscle cells, retinal epithelial cells and glomerular endothelial cells [20]. Upregulation of VEGF-A has been demonstrated in the retina of diabetic patients and experimental rat models of type 1 and type 2 diabetes [21]. The cause of VEGF-A upregulation in diabetes remains speculative, but multiple factors may be implicated. Several factors relevant to the pathogenesis of diabetic complications have been shown to promote VEGF-A expression in various cell types and tissues, including angiotensin II, reactive oxygen species, and transforming growth factor-beta (TGF-β) [22]. Taken together, it is important to consider the interaction of different pathways which could act independently or in combination to increase VEGF-A expression in the diabetic kidney. However, the relevance of these pathways remains to be determined. In addition to increased VEGF-A expression, a disturbed feedback regulation of VEGF-A could contribute to the pathophysiologic effects of VEGF-A in the diabetic kidney [23].

Endostatin is a naturally-occurring 20-kDa C-terminal fragment derived from type XVIII collagen. It is reported to serve as an anti-angiogenic agent and may interfere with the pro-angiogenic action of growth factors such as VEGF-A [24]. Endostatin was first found secreted in the media of non-metastasizing mouse cells from a hemangioendothelioma cell line in 1997 and was subsequently found in humans [25]. It suppresses angiogenesis through several pathways affecting both cell viability and movement [26].

It is shown in the present study, in high glucose *in vitro*, silencing VEGF-A in podocytes and NRK-52E cells caused upregulation of phosphorylated Thr^308^-AKT. This effect resulted in an increased nephrin expression in podocytes and decreased Ang II and AT1R expressions in NRK-52E cells. In either cell type, VEGF-A silencing caused no effects on levels of total AKT and phosphorylated Ser^473^-AKT, while AKT inhibition with MK-2206 significantly upregulated VEGF-A expression. These results demonstrated that AKT signaling pathway was involved in regulating VEGF-A activities in podocytes and NRK-52E cells. *In vivo*, VEGF-A was upregulated in both glomeruli and tubules of diabetic rats compared with normal control rats at 12 weeks after streptozotocin induction. In microdissected glomeruli, the levels of phosphorylated Thr^308^-AKT and nephrin were decreased after VEGF-A upregulation and were rescued with the endostatin treatment. However, there were no changes in the levels of total AKT and phosphorylated Ser^473^-AKT. Phosphorylated Thr^308^-AKT colocalized with nephrin in podocytes, suggesting their interaction in the regulation of glomerular VEGF-A activity in experimental type 1 diabetes. In microdissected diabetic renal tubules, with the increased VEGF-A expression, levels of Ang II and AT1R were increased and phosphorylated Thr^308^-AKT was decreased. These activities were rescued with the endostatin treatment. There were no changes in levels of total AKT and phosphorylated Ser^473^-AKT with or without the endostatin treatment. Phosphorylated Thr^308^-AKT colocalized with Ang II in renal tubules, suggesting their interaction in regulating tubular VEGF-A activity in type 1 diabetes.

Based on the *in vivo* studies, an adequate serum VEGF-A inhibitory activity was achieved during the entire course of endostatin treatment, which prevented early renal dysfunction in diabetic rats. These findings are consistent with a previous report that blockade of VEGF-A signaling ameliorates diabetic albuminuria in mice [27]. The present results indicate that VEGF-A upregulation in the diabetic kidney contributes to the pathophysiology of early renal dysfunction. The glomerular VEGF-A upregulation induced downregulation of nephrin and AKT phosphorylation, which, in part, caused podocyte injury and loss. With the endostatin treatment, podocyte injury and renal function were improved, suggesting a critical role for antiangiogenic treatment in the early stages of experimental diabetes.

We found that in diabetic rats tubulointerstitial fibrosis preceded the onset of glomerular injuries, as was shown with Masson’s trichrome staining. The reason could be due to the vulnerability of tubulointerstitium to injuries under hyperglycemic and hypoxic conditions compared with glomeruli. In NRK-52E renal tubular epithelial cells cultured in high glucose, fibronectin was upregulated (data not shown), suggesting a possible pathway that VEGF-A upregulation initiated early tubulointerstitial fibrosis.

The effect of VEGF-A inhibition does not exclude the involvement of other growth factors in the pathogenesis of early diabetic renal dysfunction. In the present study, administration of endostatin did not prevent significant mesangial expansion in diabetic rats, indicating that the degree of compensatory angiogenesis must be low in this model. It could also be caused by the inhibition of other factors, such as TGF-β and the growth hormone/insulin-like growth factor-I (IGF-I) system, resulting in the manifestations of mesangial changes not fully shown in these early experimental diabetic rats.

## Conclusions

In conclusion, blockade of VEGF-A by endostatin has renoprotective effects in a rat model of early streptozotocin-induced diabetes. The present study demonstrates a new mechanism that increased VEGF-A expression in glomeruli and renal tubules causes renal dysfunction through AKT dysregulation. The results suggest a role for constitutive VEGF-A in protecting the kidney from injurious effects, such as hyperglycemia, which might have important implications for the future development of VEGF-A inhibitory strategies for the treatment of early diabetic nephropathy.
