# Correction: Antiangiogenic Treatment Diminishes Renal Injury and Dysfunction via Regulation of Local AKT in Early Experimental Diabetes

**DOI:** 10.1371/journal.pone.0103926

**Published:** 2014-07-29

**Authors:** The PLOS ONE Staff

There are errors in the legends of Figures 1, 3, and 4. Please read the scale bar ‘50 µm' instead of ‘50 mm' in Figure Legends 1, 3, and 4. The authors have provided the correct versions of Figure Legends 1, 3, and 4 here.

**Figure 1. VEGF-A siRNAs cause upregulation of phosphorylated Thr^308^-AKT and nephrin in podocytes in high glucose.** (A) Upregulated pAKT (T308) while (B) no effects on pAKT (S473) in podocytes mediated by VEGF-A_siRNA#1 and #2 (immunofluorescence, scale bar 50µm). (C) Upregulated nephrin mRNA while no effects on total AKT mediated by VEGF-A_siRNA#1 and #2 as shown in quantitative real time RT-PCR. (D) Upregulated protein levels of pAKT (T308) and nephrin, while no effects on total AKT and pAKT (S473) mediated by VEGF-A_siRNA#1 and #2, as shown in Western blot analysis and (E) the relative quantification normalized by β-actin expression (*P<.05 vs. NT_siRNA). VEGF-A: vascular endothelial growth factor-A; pAKT (T308): phosphorylated Thr^308^-AKT; pAKT (S473): phosphorylated Ser^473^-AKT; NT_siRNA: non-targeting small interference RNA.

**Figure 3. MK-2206 inhibits AKT and upregulates VEGF-A in podocytes.** (A) Upregulated VEGF-A and downregulated pAKT (T308) and (B) pAKT (S473) in podocytes with MK-2206 treatment (immunofluorescence, scale bar 50µm). (C) Inhibition of pAKT (T308) and pAKT (S473) and increased VEGF-A by MK-2206 as shown in Western blot analysis and (D) the relative quantification normalized by β-actin expression (*P<.05 vs. vehicle). DMSO: dimethyl sulfoxide; pAKT (T308): phosphorylated Thr^308^-AKT; pAKT (S473): phosphorylated Ser^473^-AKT.

**Figure 4. MK-2206 inhibits AKT and upregulates VEGF-A in NRK-52E cells.** (A) Upregulated VEGF-A and downregulated pAKT (T308) and (B) pAKT (S473) in NRK-52E cells with MK-2206 treatment (immunofluorescence, scale bar 50µm). (C) Inhibition of pAKT (T308) and pAKT (S473) and increased VEGF-A by MK-2206 as shown in Western blot analysis and (D) the relative quantification normalized by β-actin expression (*P<.05 vs. vehicle). pAKT (T308): phosphorylated Thr^308^-AKT; pAKT (S473): phosphorylated Ser^473^-AKT.
